# The Wide Spectrum of COVID-19 Clinical Presentation in Children

**DOI:** 10.3390/jcm9092950

**Published:** 2020-09-12

**Authors:** Nadia Nathan, Blandine Prevost, Chiara Sileo, Nicolas Richard, Laura Berdah, Guillaume Thouvenin, Guillaume Aubertin, Thibault Lecarpentier, Aurélie Schnuriger, Julien Jegard, Isabelle Guellec, Jessica Taytard, Harriet Corvol

**Affiliations:** 1 Pediatric Pulmonology Department, APHP Hôpital Trousseau, Sorbonne Université, 75012 Paris, France; nadia.nathan@aphp.fr (N.N.); blandine.prevost@aphp.fr (B.P.); nicolas.richard@aphp.fr (N.R.); laura.berdah@aphp.fr (L.B.); guillaume.thouvenin@aphp.fr (G.T.); guillaume.aubertin@aphp.fr (G.A.); jessica.taytard@aphp.fr (J.T.); 2 Sorbonne Université, Inserm UMR_S933, Childhood Genetic Disorders, 75012 Paris, France; 3 Pediatric Radiology Department, APHP Hôpital Trousseau, Sorbonne Université, 75012 Paris, France; chiara.sileo@aphp.fr; 4 Sorbonne Université, Centre de Recherche Saint-Antoine (CRSA), Inserm UMR_S938, 75012 Paris, France; aurelie.schnuriger@aphp.fr; 5 Pediatric Emergency Department, APHP Hôpital Trousseau, Sorbonne Université, 75012 Paris, France; thibault.lecarpentier@aphp.fr; 6 Virology Department, APHP Hôpital Trousseau, Sorbonne Université, 75012 Paris, France; 7 Pediatric and Neonatal Intensive Care Unit, APHP Hôpital Trousseau, Sorbonne Université, 75012 Paris, France; julien.jegard@aphp.fr (J.J.); isabelle.guellec@aphp.fr (I.G.); 8 Sorbonne Université, Inserm UMR_S1158, Experimental and clinical respiratory neurophysiology, 75013 Paris, France

**Keywords:** COVID-19, SARS-CoV-2, children, infants, acute respiratory distress syndrome, PIMS-TS, MIS-C

## Abstract

**Background:** Ten months after its appearance in December 2019, SARS-CoV-2 has infected more than 25 million patients worldwide. Because children were first identified as potential spreaders of the virus, schools were closed in several countries. However, it rapidly became evident that the number of hospitalized children infected by SARS-CoV-2 was dramatically lower than that of adults. To date, only hypotheses have been raised to explain this difference, so it is of great importance to describe the presentation of this disease among children. Here, we describe a wide spectrum of COVID-19 manifestation in children in a dedicated pediatric unit in France. **Methods:** Patients hospitalized with COVID-19 who were diagnosed on the basis of either positive SARS-CoV-2 RT-PCR in nasopharyngeal swabs and/or typical aspects in chest-computed tomography (CT) were included between March and May 2020 in Paris. **Results:** Twenty-three patients were included on the basis of positive RT-PCR (*n* = 20) and/or typical aspects in CT (*n* = 4). The median age was 4.9 years [0.1–17.6]. Patients were grouped by age (<2 years old: *n* = 14, 61%; 2–10 years old: *n* = 2, 9%; >10 years old: *n* = 7, 30%). Overweight or obesity was reported in only three patients. At presentation, the most frequent symptom in the overall cohort was fever (*n* = 18, 78%), followed by acute rhinitis (*n* = 9, 64%) and cough (*n* = 7, 50%) in the under 2-year-old group and cough (*n* = 4, 57%), fatigue, dyspnea and abdominal pain (*n* = 3, 43% each) in the over 10-year-old group. Five patients required ICU treatment, four of whom were aged >10 years, two presented with acute myocarditis, and two were sickle cell disease patients who presented with acute chest syndrome. **Discussion and conclusion:** The youngest patients seem to present milder forms of COVID-19 without the need for ICU treatment and with a shorter length of hospitalization. More severe evolutions were observed in teenagers, with, however, favorable outcomes. Given the context of closed schools and confinement, the infection of these children suggests intra-familial transmission that needs to be further assessed. This description might help to understand the intriguing differences in COVID-19 severity across age-classes.

## 1. Introduction

Since December 2019, the coronavirus disease 2019 (COVID-19) outbreak has drastically changed the world’s health concerns. As of 31st August 2020, more than 25 million patients have been infected with the new severe acute respiratory syndrome coronavirus 2 (SARS-CoV-2), which spread from a Chinese cluster to almost all other countries within a few weeks [1]. Given the common knowledge that respiratory viruses, including viruses from the Coronaviridae family, usually affect more children than adults and that social distancing is difficult to apply to children, most countries decided to close their schools as the very first step in attempts to control the outbreak. The immediate effect was the emergence of a great fear of this new virus among populations.

After a few months of the virus disseminating around the world, official epidemiological data from the infected countries were published, as well as literature reports from medical teams in charge of COVID-19 patients. Even though there are only a few explanations for this observation to date, it rapidly became evident that the proportion of infected children, either symptomatic or not, was dramatically lower than that of adults [2]. Moreover, children harbored far less severe forms of the disease [3,4,5]. Indeed, incidence curves of hospitalized patients increase with age, with most severe cases being in adults over 80 years old. Mortality follows this trend and mainly burdens the elderly, but rarely children [6]. Thus, children are a minority of hospitalized patients [7]. Given the limited number of diagnosed pediatric cases and the low associated morbidity in children, the literature on pediatric COVID-19 cases is relatively scarce in comparison with reports on affected adults.

However, as confinement measures are being lifted worldwide, discussions are arising on the best ways to reopen schools and to organize the beginning academic session. Thus, a more thorough description and better understanding of COVID-19 in children are urgently needed. As a specialized COVID-19 pediatric unit, we aim to report here the intriguing repartition and presentation of COVID-19 in hospitalized children.

## 2. Materials and Methods

Following the spread of the COVID-19 outbreak in France, especially in the Paris area, our pediatric pulmonology department in the university hospital Trousseau, Assistance Publique Hôpitaux de Paris (APHP), was adapted to a COVID-19 pediatric unit in mid-March 2020. All patients with a suspected case of COVID-19 who required hospitalization were thus transferred from the emergency room to this dedicated COVID-19 unit and were tested by real-time reverse transcription (RT)-PCR for SARS-CoV-2 in nasopharyngeal swabs and occasionally in stools. If patients were experiencing respiratory symptoms, they also underwent thoracic imaging. When performed, thoracic high-resolution computed tomography (HRCT) or computed tomography pulmonary angiography (CTPA) was centrally analyzed following a standardized report written by the French Radiology Society [8]. The report includes the main thoracic HRCT or CTPA patterns described as being suggestive of COVID-19: ground-glass opacities (GGO), ground-glass opacities and consolidation, bilateral distribution of the lesions, round aspect of the lesions, peripheral distribution and pulmonary embolisms [9,10].

To account for the potential lack of performance of the RT-PCR for SARS-CoV-2 due to sampling quality or kinetics (sensitivity <60%) [11,12,13], a body of evidence was used for a COVID-19 diagnosis in hospitalized children between 10th March and 10th May 2020: (i) a positive SARS-CoV-2 RT-PCR test in nasopharyngeal swabs and/or (ii) typical aspects of COVID-19 in HRCT or CTPA.

Patient information was retrieved from medical records, including COVID-19 transmission history; clinical, biological (blood tests, viral RT-PCR findings) and radiological information; and medical evolution. After the patient was discharged, a dedicated pediatrician supervised the follow-up, which consisted of a daily phone call or online video consultation using a standardized questionnaire for 2 weeks after the onset of symptoms or until complete recovery.

The study was approved by the local ethics committee of our institution, which waived the need for patient consent (study PED_COVID N°20200717191204). The physician in charge of the patient described the study to patients and/or their legal guardians and obtained their permission. Data were extracted from electronic medical records and de-identified. Descriptive statistics are used for all study variables. Continuous data are expressed as median and range values, while categorical data are expressed as numbers and proportions (%).

## 3. Results

### 3.1. Clinical Characteristics at COVID-19 Onset

**Figure 1:**
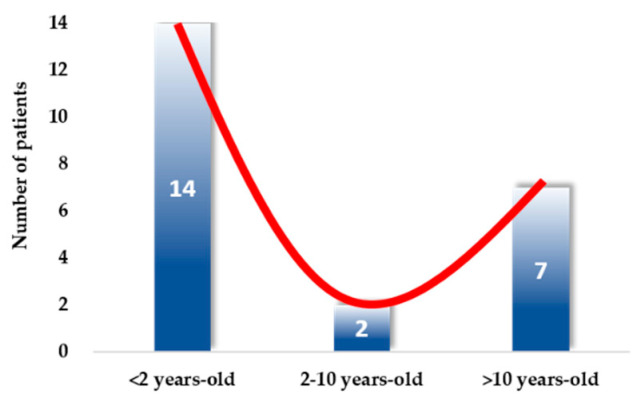
Distribution of children with COVID-19 per age-class. Among the 23 included patients, the majority (*n* = 14, 61%) were aged under 2 years old, only 9% (*n* = 2) were aged 2–10 years old, and 30% (*n* = 7) were over 10 years old. The red line illustrates the distribution across age-classes.

For 2 months, between March 10th and May 10th, 2020, 23 children were hospitalized in our dedicated COVID-19 unit. The majority of the patients were infants younger than 2 years old (*n* = 14, 60.8%); very few were between 2 and 10 years old (*n* = 2, 8.7%), and the others were older than 10 years old (*n* = 7, 30.4%) (Figure 1 and Supplemental Figure S1). As described below, COVID-19 diagnosis was confirmed by positive RT-PCR for SARS-CoV-2 in nasopharyngeal swabs for 20 children (including two children who were also positive for SARS-CoV-2 by RT-PCR in stools) and by chest HRCT or CPTA for three children older than 10 years old who were negative for SARS-CoV-2 by RT-PCR in nasopharyngeal swabs (two of them were also positive for SARS-CoV-2 by serology, and the third one lived with an adult (his mother) who was diagnosed positive for SARS-CoV-2 by RT-PCR in a nasopharyngeal swab).

Clinical characteristics of the 23 patients are described in Table 1 by age-class, i.e., under 2 years old, between 2 and 10 years old, and over 10 years old. The sex ratio was roughly equally distributed in the overall cohort, with a male predominance in the youngest group (64%). The majority (65%) of the patients had at least one parent from an ethnic minority group, with 43% from Sub-Saharan African and Caribbean ancestries (detailed in Table 1). Ten patients (43.5%) were previously healthy, but 13 (56.5%) had preexisting comorbidities (including all patients but one over 10 years old). The most frequent comorbidities were asthma (*n* = 3), sickle cell diseases (SCD, *n* = 3) and overweight or obesity (*n* = 3) (detailed in Table 1). None of them received non-steroidal anti-inflammatory drugs prior to admission [14,15,16,17]. Of importance, 20 (87%) patients, including 100% of the infants, cohabitated with an adult with clinical signs suggestive of a viral infection in the days prior to the onset of the symptoms of the child.

At presentation, the most frequent symptom in the overall cohort was fever (*n* = 18, 78%), followed by acute rhinitis (*n* = 9, 64%) and cough (*n* = 7, 50%) in the under 2-year-old group and by cough (*n* = 4, 57%), fatigue, dyspnea and abdominal pain (*n* = 3, 43% each) in the over 10-year-old group. Finally, neurologic symptoms were found in 5 (22%) children overall. Only one patient of this series, aged 17, reported anosmia (Table 1). Co-viral infections were found in five children, among whom four had a positive SARS-CoV-2 RT-PCR result, and one had a HRCT scan that showed typical aspects associated with positive SARS-CoV2 serology. Three out of these five children were under 2 years old and were co-infected with adenovirus (*n* = 1), bocavirus (*n* = 1) and rhinovirus plus adenovirus (*n* = 1). The two remaining patients, aged 5 and 12, were co-infected with Epstein–Barr virus.

Blood test results, described in Table 2, were normal in most patients, except for the patients who developed acute myocarditis (*n* = 2) as well as those with SCD who developed acute chest syndrome (*n* = 2) (Supplemental Figure S2). Indeed, the patients who developed acute myocarditis, aged 5 and 11 years old, had very high levels of C-reactive protein (CRP: 183 and 315 mg/L, respectively) and elevated liver enzymes (AST: 99 and 77 IU/L, respectively). The older patient also had lymphopenia (0.4 × 10^9^/L) (Supplemental Figure S2A). The two patients with SCD who developed acute chest syndrome, aged 16 and 17 years old, also showed elevated levels of CRP (329 and 66 mg/L, respectively), as well as anemia (8.2 and 7.5 g/dL, respectively) (Supplemental Figure S2B).

Chest X-rays were performed for 19 children, either because they experienced respiratory symptoms or as part of a systematic evaluation in infants under 3 months with fever. The results were normal for 10 (53%) patients and showed non-specific abnormalities for the others, such as hyperinflation (*n* = 2), bronchial thickening (*n* = 1), pleural effusion (*n* = 1) and/or alveolar consolidations (*n* = 6). Among the latter, two patients (one in the 2-10-year-old group and one in the over 10-year-old group) had retrocardiac consolidations with no lower respiratory signs at clinical examination, but abdominal pain was at the forefront of the clinical presentation. Chest HRCT or CPTA was performed in 4 (17%) patients aged 11.3–16.6 years old. The results were suggestive of COVID-19 for all of them. The main HRCT and CTPA findings were ground-glass opacities and consolidation (*n* = 4); bilateral distribution of the lesions (*n* = 4), with a predominant round aspect of the lesions in one patient; peripheral distribution (*n* = 3); peripheral and central distribution (*n* = 1); and pulmonary embolism (*n* = 1).

### 3.2. Treatments and Evolution

**Figure 2:**
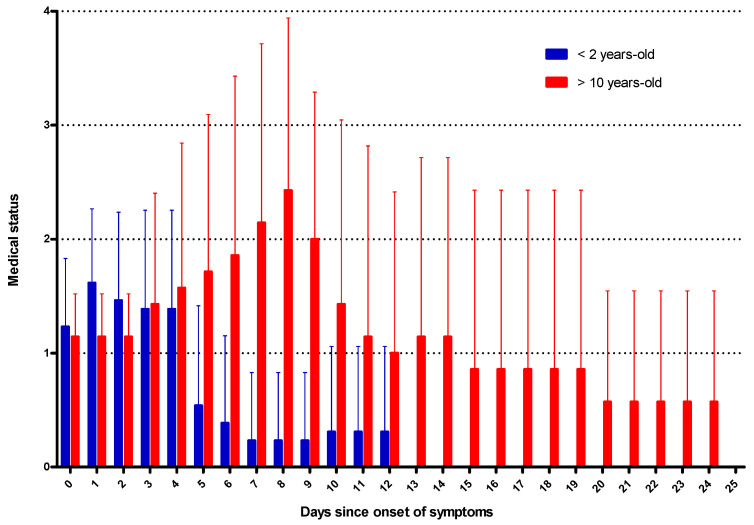
Graphical timeline of the clinical evolution of the 21 children with symptomatic COVID-19. Medical status was determined daily for each patient from the onset of symptoms (day 0) until discharge home and scored from 0 to 4: 1 = presence of symptoms, 2 = hospital admission, 3 = oxygen therapy, 4 = ventilatory support (non-invasive ventilation (NIV) or mechanical ventilation) and 0 = discharge. The data are the mean of the daily medical status scores in each age group (<2 years old in blue and >10 years-old in red) from the onset of symptoms. Error bars show standard deviations. As only one patient showed symptoms in the 2–10-year-old group, this age group is not represented in this figure. This figure illustrates that in the youngest patients, the disease is symptomatic at the onset but has a rapid positive outcome, whereas, in older children, the severity subsequently increases, sometimes requiring intensive care and longer hospitalizations.

The global evolution patterns of COVID-19 for children under 2 years old and over 10 years old are represented in Figure 2. This figure illustrates that, while the disease is symptomatic from the outset in the youngest patients, the evolution is quickly favorable, whereas, in older children, it subsequently becomes more severe and requires longer hospitalizations.

#### 3.2.1. Infants (<2 Years Old, *n* = 14)

None of the 14 infants (the under 2-year-old group) required admission to the intensive care unit (ICU) for COVID-19 (Table 3). Interestingly, one 2-month-old boy with a complex immune deficiency was previously hospitalized in the ICU for acute respiratory distress syndrome (ARDS) associated with invasive aspergillus infection. After ICU discharge, he was referred to a medical unit for 10 days. During this hospitalization, his mother developed clinical symptoms of COVID-19 and was further confirmed to be positive for COVID-19. She was in close contact with the patient and probably transmitted the virus to him. COVID-19-related symptoms remained limited in this infant, who did not develop secondary ARDS despite his immune deficiency. All other infants with SARS-CoV-2 infection had a very favorable outcome, as we previously described in a subset of the youngest patients [18]. They received no drugs other than acetaminophen and were discharged home after a median of 3 days of hospitalization.

#### 3.2.2. Children (2–10 Years Old, *n* = 2)

One patient was a 5.9-year-old girl of African ancestry with a medical history of obesity (Body Mass Index (BMI) Z-score 3.8) and febrile seizures. She presented with acute and febrile abdominal pain that led to an appendicectomy. After surgery, a persistent high fever led to the diagnosis of pericarditis and acute myocarditis associated with pediatric inflammatory multisystem syndrome temporally associated with SARS-CoV-2 infection (PIMS-TS), further complicated by hemodynamical impairment with rash and drowsiness (Table 3 and Supplemental Figure S2A). She presented with moderate anemia with inflammatory syndrome with mild elevation of liver enzymes. She required 5 days of ICU care, 3 days of mechanical ventilation, intravenous immunoglobulins, antibiotics and inotropic support (but no corticosteroids or convalescent plasma). The evolution was satisfactory, and she was discharged home without sequelae after 12 days of hospitalization. The second patient of this age-class was hospitalized for a severe household burn. He tested positive for SARS-CoV-2 because one of his parents developed the disease in the previous weeks. He remained asymptomatic during the entire duration of his hospitalization (11 days).

#### 3.2.3. Teenagers (>10 Years Old, *n* = 7)

Among the seven children older than 10 years old, four (57%) required ICU treatment for ARDS. All were of African ancestry. Two of them were girls aged 16 and 17 years-old with SCD who also presented with acute chest syndrome (Supplemental Figure S2B). Both presented with an inflammatory syndrome together with anemia and focal alveolar opacities in chest X-rays or CT scans, suggestive of acute chest syndrome. One of them was complicated by pulmonary embolism. They required 6 and 4 days of non-invasive ventilation, respectively, antibiotics and red blood cell exchange transfusions. For the most severe patient, aged 16 years, who also had pulmonary embolism, anticoagulation and Tocilizumab were added to her supportive care, and she improved rapidly, as we previously reported in Odièvre et al. [19]. Interestingly, none of the patients were put on any therapy being tested in COVID-19 clinical trials in adults such as antiviral, hydroxychloroquine, azithromycin or convalescent plasma.

The third patient with ARDS had obesity (BMI Z-score 3.9) and epilepsy related to a rare disease called myoclonic-astatic epilepsy. After admission to our unit, he rapidly developed ARDS, which prompted us to perform HRCT, and the results were typical of COVID-19, with lobar consolidations and ground-glass opacities. Despite the negativity of the SARS-CoV-2 RT-PCR in nasopharyngeal swabs, he had positive SARS-CoV-2 serology. He developed neurological symptoms, such as drowsiness, but the cerebrospinal fluid sample analysis was normal and negative for SARS-CoV-2 by RT-PCR. He was admitted to the ICU but did not require respiratory support other than oxygen therapy. He was discharged home after 5 days of hospitalization (Table 3).

The fourth patient who required ICU admission was an 11-year-old girl who was overweight (BMI Z-score 2.6). She was first suspected of appendicitis with high fever and abdominal pain, which led to an appendectomy, similar to the patient reported in the previous section. The fever remained afterward, together with acute headaches and vomiting, as she developed acute PIMS-TS (Table 3 and Supplemental Figure S2A). Her biological analyses revealed an inflammatory syndrome with mildly elevated liver enzymes and lymphopenia. She required 5 days of mechanical ventilation, intravenous immunoglobulins, antibiotics and inotropic support (but no corticosteroids or convalescent plasma). The evolution was satisfactory, and she was discharged home without sequelae after 21 days of hospitalization.

Three out of the seven patients aged over 10 years did not require ICU treatment: (i) one patient was systematically tested for SARS-CoV-2 before an appendicectomy but presented no COVID-19-related symptoms; (ii) the second one, who had a medical history of bronchiectasis and severe allergic asthma, presented with an asthma attack associated with acute abdominal pain and required oxygen therapy for one day; (iii) the third one had a medical history of severe post-infectious obstructive disease and presented with a mild form of COVID-19 with fever, cough and wheezing, but no oxygen was required.

## 4. Discussion

In this study, we describe a wide spectrum of COVID-19 expression in children. For 2 months, between 10th March and 10th May 2020, 23 children with COVID-19 were admitted to our dedicated pediatric unit. Interestingly, we observed a peculiar distribution: a larger proportion of hospitalized children were under 2 years old, and most severe patients were above 10 years old.

### 4.1. COVID-19 Severity is Inversely Correlated with Age

Two main conclusions arise from these observations: the youngest patients had the mildest form of COVID-19 and few preexisting comorbidities, whereas the oldest ones had a greater proportion of preexisting comorbidities and developed more severe forms. Infants represented the main fraction of patients in this study. They were mainly hospitalized because of their young age (most of them were under 3 months) and poorly tolerated fever. Their respiratory symptoms were mild and the evolution was rapidly favorable, as we previously described [18]. None of them required ICU management or prolonged hospitalization (median length: 3 days, extremes 1–7).

On the other hand, teenagers (children over 10 years old) also represented a large part of the study population. Unlike the infants, they displayed a more severe presentation and evolution with respiratory and/or abdominal symptoms at the forefront. More than half of them required ICU admission for ARDS (high-flow oxygen support, non-invasive or even mechanical ventilation) and/or for hemodynamic failure. Interestingly, two of them had SCD. Furthermore, the association between acute chest syndrome in patients with SCD and SARS-CoV-2 infection is intriguing. Indeed, the high number of similar cases in the literature so far suggests a poor tolerance of COVID-19 in patients with SCD, probably due to the SCD vasculopathy that worsens SARS-CoV-2 pathogenicity in the lungs [19,20,21,22,23]. However, in the described teenagers, despite the severe initial presentation, even when burdened by a preexisting chronic disease such as SCD, obesity or epilepsy, no fatal evolution was observed in this pediatric series, and all of the patients recovered without any sequelae [22,24]. As few patients were aged between 2 and 10 years, the overall severity of this age group was harder to assess.

### 4.2. Pediatric Inflammatory Multisystem Syndrome Linked to SARS-CoV-2 Infection

Only two patients of this series presented with a hyperinflammatory syndrome and multiorgan involvement, recently named PIMS-TS in Europe [25] and “multisystem inflammatory syndrome in children” (MIS-C) in the US [26]. Interestingly, both presentations were quite similar, with acute abdominal pain and fever that led to a false diagnosis of appendicitis (and appendectomy) before the diagnosis of PIMS-TS was confirmed. Several studies have described this aberrant immune reaction to SARS-CoV-2 infection, with excessive inflammation leading to acute heart failure [27,28,29,30,31,32], which had previously been reported for other viruses, such as Coronavirus 229E [33].

In the UK, a very recent multicenter observational study described 78 children admitted to the ICU for PIMS-TS [29]. The median age was 11 years [8–14], 67% were male, and patients with ethnic minority backgrounds were over-represented (78%). The majority of the patients presented with at least one abdominal symptom (i.e., abdominal pain, diarrhea, vomiting), and the other common presenting features were fever and shock. SARS-CoV-2 infection was confirmed either by positive RT-PCR and/or by IgG serology. Treatments included mechanical ventilation, vasoactive infusions, steroids and intravenous immunoglobulin. Three children needed extracorporeal membrane oxygenation, and two children died. In France, an observational study reported 21 children with this multisystem inflammatory syndrome, among whom 12 presented with Kawasaki disease shock syndrome, 16 had myocarditis, and 19 had a proven recent SARS-CoV-2 infection [32]. The median age of this series was 7.9 years [3.7–16.6], and a high frequency of children from African ancestry was reported. The main symptoms were also gastrointestinal, associated with hemodynamic instability and myocarditis. With a focus on the cardiac consequences, a study performed in centers from France and Switzerland reported that 35 children (median age: 10 years [2–16]) presented with cardiogenic shock, left ventricular dysfunction and a severe inflammatory state [27]. Among these children, 31 tested positive for SARS-CoV-2 (RT-PCR or serology). Similar to our study, comorbidities were present in one-third of the patients, including asthma and overweight. Interestingly, a French pilot study evaluated cardiac Magnetic Resonance Imaging (MRI) in four patients with PIMS-TS. MRI indicated post-infectious myocarditis, showing diffuse myocardial edema, with no evidence of replacement fibrosis or focal necrosis, which is usually observed in acute viral myocarditis [28].

### 4.3. In Infants, COVID-19 Seems to Lead to a Mild Respiratory Disease

We were surprised to observe that infants who developed COVID-19 presented no or limited signs of respiratory distress, whereas adults with symptomatic SARS-CoV-2 infection frequently show respiratory signs at the forefront. However, this raises questions, as infants are usually more vulnerable to respiratory viral infections than adults. In winter, pediatric hospitals need to increase their hospitalization capacities to admit infants with acute bronchiolitis, a viral infection of the lower respiratory tract. Among the viruses associated with bronchiolitis, respiratory syncytial virus (RSV) remains the most frequent, but other viruses are also identified, such as rhinovirus, metapneumovirus, bocavirus, (para)influenza virus or adenovirus [34]. Coronaviruses are also associated with bronchiolitis, but these are different species from SARS-CoV-2, i.e., 229E, NL63, OC43 and HKU1 [35,36]. The symptoms of bronchiolitis include cough, tachypnoea, fever and feeding difficulties, and this frequent disease sometimes leads to ARDS requiring ICU care. Acute bronchiolitis remains one of the most important health burdens for infants and young children worldwide. Thus, one major question is raised: given that infants can be infected with SARS-CoV-2, why are their respiratory diseases much less severe compared with adults [37,38,39]?

While some authors have argued that children might be less susceptible to SARS-CoV-2, a retrospective analysis of a large pediatric cohort with COVID-19 in China supports the evidence that children are as susceptible as adults to this infection [40]. Therefore, a second major question is raised: since children seem to be as susceptible as adults to SARS-CoV-2 infection, why are their diseases much less severe? Two hypotheses can be raised. The first one is that SARS-CoV-2 has lower pathogenicity in the youngest patients [41]. Various explanations have been offered, including a lower response of the epithelial cell receptors to SARS-CoV-2 and innate and adaptive immune differences between children and adults [42,43]. The second hypothesis is that, while the pathogenicity is similar in children and adults, children might be less severely infected because of social determinants of health and government actions, such as daycare and school closures and confinement.

### 4.4. Immune and Epithelial Response to SARS-CoV-2 in Children

Innate and adaptative responses to SARS-CoV-2 have been suggested as possible “protective” factors against SARS-CoV-2 infection in children. Indeed, memory cells allow the immune system to provide a quicker and stronger response to a viral re-infection, further providing cross-protection against other viruses. Vaccinations can also enhance this “trained” innate immune response toward pathogens beyond those that are specifically targeted by the vaccine [44]. In comparison with adults, children are both highly infected by viruses and subject to various repeated vaccinations, which may account for the role of this trained immunity in the puzzling resistance of children to COVID-19 [39]. Moreover, this might explain the underrepresentation of children between 2 and 10 years old in our series, as they should be the group the benefits the most from trained immunity since they are both in contact with lots of viruses and largely vaccinated. In the young infant group, trained immunity may not yet be effective, as vaccinations are just starting at this age (mostly scheduled after 2–3 months old) and as contact with childhood viruses is limited (young infants are mostly cared for at home by their mothers on maternity leave). The teenaged group was the group of children that developed the most severe cases of COVID-19 in our series as well as in series reported by others worldwide [40]. They are indeed less exposed to viruses compared with younger children and are further away from the intense vaccination period. Thus, they could be assimilated to “young adults” in terms of the SARS-CoV-2 immune response.

It has also been suggested that the milder COVID-19 expression in children could be related to a differential expression of the cell receptor for SARS-CoV-2, angiotensin-converting enzyme II (ACE2) [45,46,47]. It was recently shown that ACE2 nasal expression was age-dependent: it is lowest in younger children and increases with age [48]. It was also reported that ACE2 expression was downregulated by several types of coronaviruses, i.e., Human Coronavirus NL63 (HCoV-NL63), SARS-CoV and SARS-CoV-2 [49,50]. Further studies are needed to fully explore the role of ACE2 in COVID-19 expression.

### 4.5. Probable Adult-to-Children Transmission of the Virus

Family confinement and the finding that 87% of the patients cohabitated with an adult who had a suspected infection led to the inference that the majority of SARS-CoV-2 transmission was intrafamilial and vertical from an adult to the child. As countries worldwide are questioning whether schools should entirely reopen, this observation is of major importance. Fears raised against the school reopening involve, of course, the possible infection of the children but also, above all, the transmission risk to the supervising adults. This study, together with other published ones, suggests that there is a very low risk of children-to-adult transmission [51,52]. It is still unclear whether infected children can spread the virus to adults. An intriguing example is a cluster of cases in the French Alps at the beginning of the French outbreak. In this cluster, a child of 9 years attended three different schools as well as a skiing class while showing symptoms of COVID-19 but infected nobody [53]. Therefore, the major risk, in workplaces as well as in public areas, remains the adult-to-adult transmission that should be avoided by strict compliance with barrier measures between adults.

## 5. Conclusions

This study describes the wide spectrum of COVID-19 expression in children. In line with the literature, our results show that children have a much milder disease compared with adults, with discrepancies between infants and teenagers. We observed a large proportion of infected infants who improved rapidly. The older children might develop more severe disease, especially when they have preexisting comorbidities. Nevertheless, none of the children of this series died, and all recovered their preexisting clinical state.

## Figures and Tables

**Table 1:** Clinical characteristics of the 23 children with SARS-CoV-2 infection.

Clinical Characteristics	Total *n* = 23	<2 Years Old *n* = 14 (61%)	2–10 Years Old *n* = 2 (9%)	>10 Years Old *n* = 7 (30%)
Age: years; mean [range]	4.9 [0.1; 17.6]	0.4 [0.1; 1.2]	5.2 [4.5; 6.0]	14.0 [11.4; 17.6]
Gender: boys, *n* (%)	13 (57%)	9 (64%)	1 (50%)	3 (43%)
**Ancestry, *n* (%)**				
Asia	1 (4%)	1 (7%)	0 (0%)	0 (0%)
Europe	8 (35%)	5 (36%)	1 (50%)	2 (29%)
Middle East and Maghreb	4 (17%)	3 (21%)	0 (0%)	1 (14%)
Sub-Saharan Africa/Caribbean	10 (43%)	5 (36%)	1 (50%)	4 (57%)
**Comorbidities, *n* (%)**	**12 (52%)**	**5 (36%)**	**1 (50%)**	**6 (86%)**
Asthma	3 (13%)	2 (14%)	0 (0%)	1 (14%)
Sickle cell disease	3 (13%)	1 (7%)	0 (0%)	2 (29%)
Overweight/obesity	3 (13%)	0 (0%)	1 (50%)	2 (29%)
Other	4 (17%)	1 (7%) immune deficiency 1 (7%) premature birth		1 (14%) myoclonic-astatic epilepsy 1 (14%) Bronchiectasis 1 (14 %) COPD
**Exposure to SARS-CoV-2, *n* (%)**				
Family cluster	20 (87%)	14 (100%)	1 (50%)	5 (71%)
Other exposure	0 (0%)	0 (0%)	0 (0%)	0 (0%)
Unknown	3 (13%)	0 (0%)	1 (50%)	2 (29%)
**Initial symptoms, *n* (%)**	**21 (91%)**	**13 (93%)**	**1 (50%)**	**7 (100%)**
Fever	18 (78%)	11 (79%)	1 (50%)	6 (86%)
Fatigue/deterioration of general status	12 (52%)	8 (57%)	1 (50%)	3 (43%)
Cough	11 (48%)	7 (50%)	0 (0%)	4 (57%)
Pharyngitis (sore throat)	4 (17%)	1 (7%)	1 (50%)	2 (29%)
Dyspnea	5 (22%)	2 (14%)	0 (0%)	3 (43%)
Wheezing	4 (17%)	2 (14%)	0 (0%)	2 (29%)
Acute rhinitis	11 (48%)	9 (64%)	1 (50%)	1 (14%)
Acute anosmia/ageusia	1 (4%)	0 (0%)	0 (0%)	1 (14%)
Myalgia/arthralgia	0 (0%)	0 (0%)	0 (0%)	0 (0%)
Nausea or vomiting	3 (13%)	0 (0%)	1 (50%)	2 (29%)
Diarrhea	4 (17%)	1 (7%)	1 (50%)	2 (29%)
Abdominal pain	5 (22%)	0 (0%)	2 (100%)	3 (43%)
Abnormal cry	4 (17%)	4 (29%) moaning	0 (0%)	0 (0%)
Abnormal skin color	6 (26%)	4 (29%) mottled skin	1 (50%) diffuse rash	1 (14%) thoracic rash
Neurological signs	5 (22%)	3 (21%) hypotonia	1 (50%) drowsiness	1 (14%) drowsiness
**Initial vital signs, median [range]**				
Temperature (°C)	37.9 [36.6; 39.9]	38.0 [36.6; 38.7]	36.7 [36.6; 36.7]	38.1 [36.9; 39.9]
Respiratory rate (/min)	40 [15; 60]	46 [24; 60]	18 [18; 18]	30 [15; 60]
Oxygen saturation (%)	98 [85; 100]	99 [96; 100]	99.5 [99; 100]	97 [85; 99]
Cardiac rate (/min)	144 [64; 195]	161 [126; 195]	113.5 [106; 121]	123 [64; 155]

Abbreviations: COPD: chronic obstructive pulmonary disease. Sub-section headings are indicated in bold.

**Table 2:** Blood tests at admission for the 23 children with SARS-CoV-2 infection.

Blood Tests at Admission	Total *n* = 23 Median [Range]		<2 Years Old *n* = 14 Median [Range]		2–10 Years Old *n* = 2 Median [Range]		>10 Years Old *n* = 7 Median [Range]	
WBC (×10^9^/L)	9.5	[4.1–26.1]	9	[4.1–22.3]	16.3	[12.8–19.8]	11.8	[4.1–26.1]
Lymphocytes (×10^9^/L)	3.4	[0.4–7.2]	4.3	[1.0–7.2]	2.7	[1.5–4]	2.4	[0.4–3.5]
Neutrophils (×10^9^/L)	4.2	[0.8–22.2]	2.2	[0.8–5.6]	12.1	[7.3–16.8]	6.8	[2.2–22.2]
Hemoglobin (g/dL)	10.8	[7.5–15]	10.7	[8.5–14.7]	10.3	[8.2–12.4]	12.4	[7.5–15]
Platelets (×10^9^/L)	275.5	[122–492]	349	[123–492]	294	[210–378]	182	[122.0–474]
CRP (mg/L)	4	[0–329.7]	3.3	[0–32]	91.8	[0.0–183.5]	66	[0–329.7]
Procalcitonin (µg/L)	0.2	[0–242.4]	0.2	[0.1–14]	242.4	[242.4–242.4]	2.6	[0–63.5]
AST (IU/L)	44	[18.0–99]	45	[18.0–67]	61	[23–99]	33	[20–77]
ALT (IU/L)	18	[7–59]	36	[7.0–59]	15.5	[10–21]	12	[7–46]
CPK (IU/L)	170	[0–1024]	224	[224–224]	N/A		116	[0–1024]

Abbreviations: WBC: white blood cells; CRP: C-reactive protein; AST: aspartate transaminase; ALT: alanine transaminase; CPK: creatine phosphokinase.

**Table 3:** Evolution of the 21 children with symptomatic SARS-CoV-2 infection.

Evolution	Total *n* = 21	<2 Years Old *n* = 13	2–10 Years Old *n* = 1	>10 Years Old *n* = 7
**Symptoms prior to hospitalization**				
Duration (days): median [range]	3 [0–13]	1 [0–10]	6	3 [0–13]
**Overall hospitalization**				
Duration (days): median [range]	6 [1–21]	3 [1–7]	12	6 [2–21]
**ICU, *n* (%)**	**5 (24%)**	**0**	**1 (100%)**	**4 (57%)**
**Oxygen therapy, *n* (%)**	**4 (19%)**	**0**	**0**	**4 (57%)**
Duration (days): median [range]	1.5 [1–3]	–	–	1.5 [1–3]
**Non-invasive ventilation, *n* (%)**	**2 (10%)**	**0**	**0**	**2 (29%)**
Duration (days): median [range]	5 [4–6]	–	–	5 [4–6]
**Mechanical ventilation, *n* (%)**	**2 (10%)**	**0**	**1 (100%)**	**1 (14%)**
Duration (days): median [range]	4 [3–5]	–	3	5

Abbreviations: ICU: intensive care unit. Sub-section headings are indicated in bold.

## Supplementary Materials

The following are available online at https://www.mdpi.com/2077-0383/9/9/2950/s1, Figure S1: Age repartition of the included patients, Figure S2: Detailed clinical presentation of 4 patients.
