# Supplementary material for: The Wide Spectrum of COVID-19 Clinical Presentation in Children
Source: J Clin Med. 2020 Sep 12;9(9):2950. doi: 10.3390/jcm9092950 (PMC7564665; doi:10.3390/jcm9092950)
Supplement: Supplementary file 1 [file jcm-09-02950-s001.pdf]

Journal of
Clinical Medicine

MDPI

Supplemental data for

The Wide Spectrum of COVID-19 Clinical Presentation in Children

Nadia Nathan [1,2], Blandine Prevost [1], Chiara Sileo [3], Nicolas Richard [1,4], Laura Berdah [1,4], Guillaume Thouvenin [1,4], Guillaume Aubertin [1,4], Thibault Lecarpentier [5], Aurélie Schnuriger [4,6], Julien Jegard [7], Isabelle Guellec [7], Jessica Taytard [1,8] and Harriet Corvol [1,4*]

1 Pediatric pulmonology department, APHP Hôpital Trousseau, Sorbonne Université, 75012 Paris, France
2 Sorbonne Université, Inserm UMR_S933, Childhood genetic disorders, 75012 Paris, France
3 Pediatric radiology department, APHP Hôpital Trousseau, Sorbonne Université, 75012 Paris, France
4 Sorbonne Université, Centre de Recherche Saint-Antoine (CRSA), Inserm UMR_S938, 75012 Paris, France
5 Pediatric emergency department, APHP Hôpital Trousseau, Sorbonne Université, 75012 Paris, France
6 Virology department, APHP Hôpital Trousseau, Sorbonne Université, 75012 Paris, France
7 Pediatric and neonatal intensive care unit, APHP Hôpital Trousseau, Sorbonne Université, 75012 Paris, France
8 Sorbonne Université, Inserm UMR_S1158, Experimental and clinical respiratory neurophysiology, 75013 Paris, France.
* Correspondence: harriet.corvol@aphp.fr

Supplemental Figure 1. Age repartition of the included patients. The patients are represented by one-year intervals of age from 0 to 18 years-old. Each bar represents the number of patients in the one-year interval of age at the time of hospitalization.

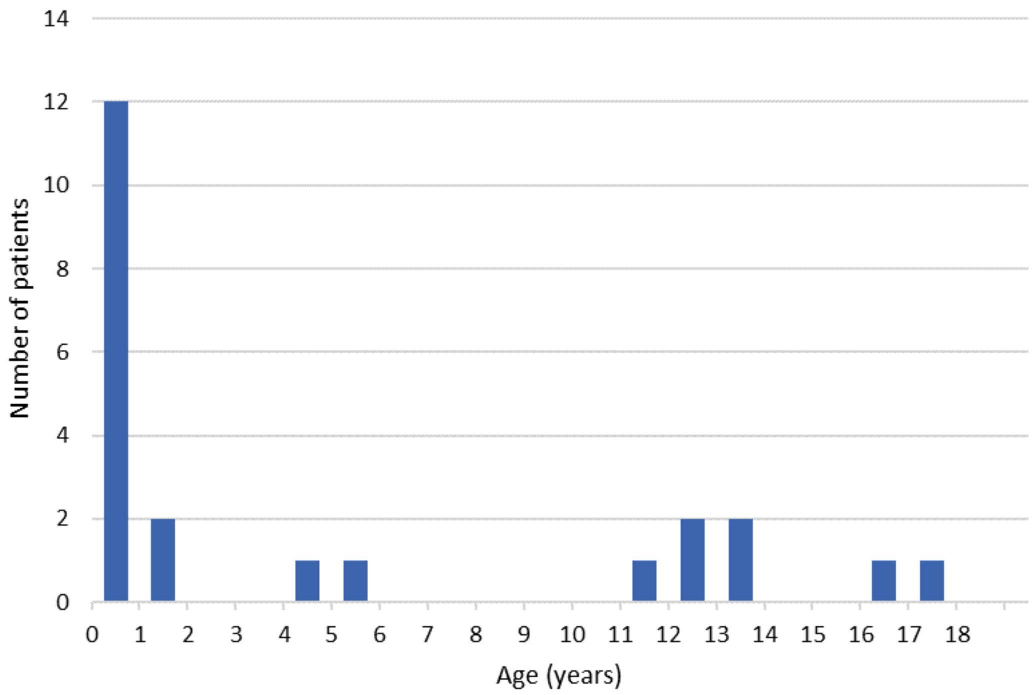

Supplemental Figure 2. Detailed clinical presentation of 4 patients. This figure depicts common (central columns) and different (side columns) clinical features for COVID-19 patients with similar presentation and comorbidities at admission. (a) **Girls aged 11 and 5 years with pediatric inflammatory multisystem syndrome temporally associated with SARS-CoV-2 infection (PIMS-TS).** Axial HRCT shows ground-glass opacities and consolidations with bilateral and peripheral distribution for the 11-year-old girl (left). The chest X-ray shows right pleural effusion and retrocardiac consolidation for the 5-year-old girl (right). (b) **Girls aged 17 and 16 with sickle cell disease (SCD) and acute chest syndrome**. The chest X-ray shows a consolidation with a round aspect for the 17-year-old girl (left). CPTA shows a segmental and subsegmental pulmonary embolism (and typical aspect of COVID-19 CT, not shown) for the 16-year-old girl (right).

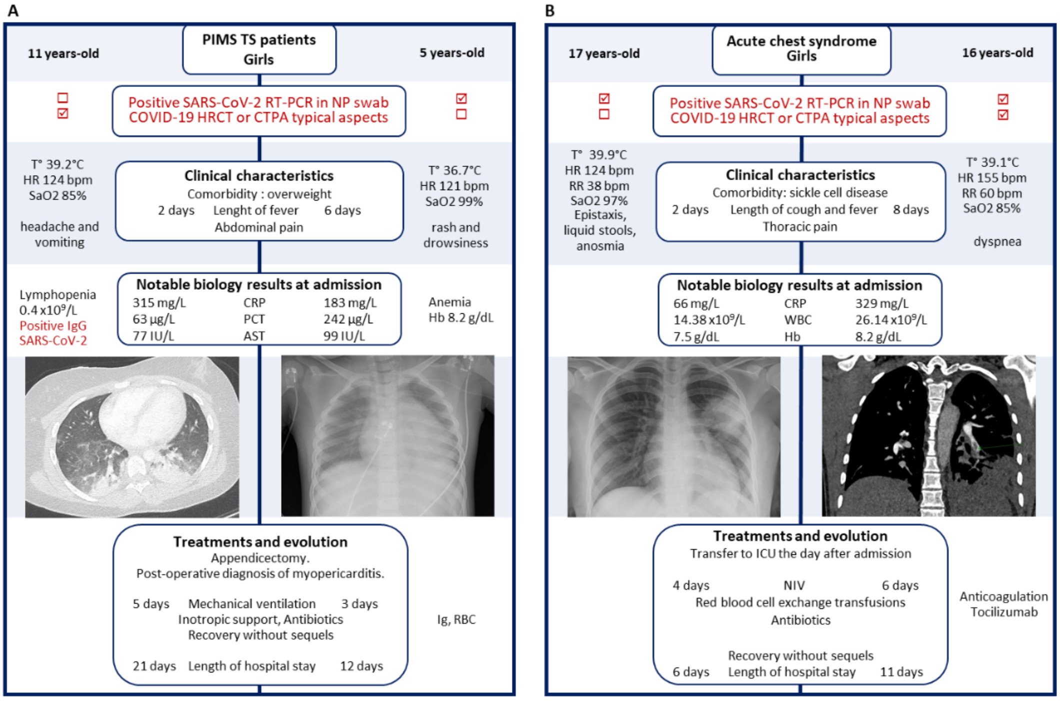

Abbreviations: PIMS-TS: pediatric inflammatory multisystem syndrome temporally associated with SARS-CoV-2 infection; RT-PCR: real-time reverse transcription PCR; SARS-CoV-2: severe acute respiratory syndrome coronavirus 2; NP: nasopharyngeal; COVID-19: coronavirus disease 2019; HRCT: high-resolution computed tomography; HR: Heart Rate; CTPA: computed tomography pulmonary angiography; RR: Respiratory rate; WBC: White blood cells; Hb: Hemoglobin; CRP: C-reactive protein; PCT: Procalcitonin; AST: aspartate transaminase; IVIG: Intravenous Immunoglobulins; RBC: Red Blood Cells; ICU: Intensive care unit; NIV: non-invasive ventilation
